# Host-Specific Induction and Functional Characterization of the Carboxylesterase *TtCarE-4* in *Tetranychus truncatus* Adaptation to Woody Host Plants

**DOI:** 10.3390/insects17090913

**Published:** 2026-09-01

**Authors:** En-Dao Wang, Zhi-Cheng Yang, Pei Wang, Cai-Li Guo, Qin-Zhe Sun, Xin Xiang, Lei Liu, Huan Liu, Sen-Shan Wang, Li-Wen Song

**Affiliations:** Biocontrol Engineering Laboratory of Crop Diseases and Pests of Gansu Province, College of Plant Protection, Gansu Agricultural University, Lanzhou 730070, China; m15593869975@163.com (E.-D.W.); 17674510183@163.com (Z.-C.Y.); 18693301779@163.com (P.W.); 13830233530@163.com (C.-L.G.); sunqinzhe926@163.com (Q.-Z.S.); weileshimo@163.com (X.X.); liulei1@gsau.edu.cn (L.L.); m15210608845@163.com (H.L.); wangsenshan@gsau.edu.cn (S.-S.W.)

**Keywords:** *Tetranychus truncatus*, host shift, carboxylesterase, detoxification enzyme system, RNAi

## Abstract

Carboxylesterases (CarEs) are detoxification enzymes that play important roles in xenobiotic metabolism and host adaptation in the polyphagous spider mite *Tetranychus truncatus*. However, the specific carboxylesterase proteins involved in host plant detoxification in *Tetranychus truncatus* remain poorly characterized. In this study, we investigated the activities of glutathione S-transferase (GST), carboxylesterase (CarE), and cytochrome P450 (CYP450) in *T. truncatus* reared on three host plants—common bean, cucumber, and jujube. CarE activity was significantly higher in mites fed on jujube, while CYP450 content increased in mites fed on cucumber. GST activity did not differ significantly among the three host plants, indicating that different hosts induce distinct detoxification responses in *T. truncatus*. Expression profiling of 24 CarE genes revealed that the carboxylesterase gene *TtCarE-4* exhibited the strongest host-specific response, with expression levels 8-fold and 160-fold higher on jujube than on bean and cucumber, respectively, consistent with changes in total CarE enzyme activity. RNAi-mediated silencing of *TtCarE-4* reduced its transcript abundance by 32.8%, significantly increased mite mortality, and decreased female fecundity. Off-target analysis indicated that the gene is specifically targeted to *T. truncatus* and is safe for non-target organisms such as natural enemies. This study provides new insights into the physiological mechanisms of host adaptation in spider mites and identifies the carboxylesterase gene *TtCarE-4* as a potential RNAi target for sustainable pest management.

## Simple Summary

Spider mites are highly polyphagous pests that frequently move between herbaceous and woody host plants in agricultural systems. Such host shifts require mites to adjust their detoxification systems to cope with different plant defense chemicals. In this study, we found that the woody host jujube specifically induced carboxylesterase activity and strongly increased the expression of the carboxylesterase gene *TtCarE-4* in *Tetranychus truncatus*. Silencing *TtCarE-4* by RNA interference significantly increased mite mortality and reduced egg production, indicating that this gene plays an important role in adaptation to woody hosts. These findings improve our understanding of host adaptation in spider mites and identify *TtCarE-4* as a promising target for RNAi-based sustainable pest management.

## 1. Introduction

*Tetranychus truncatus* is an important polyphagous agricultural pest mite, and its occurrence frequency and damage severity on various crops have been increasing in recent years [1]. This mite has a broad host range, feeding on over 120 plant species, two-thirds of which are herbaceous plants. It infests legumes, cucurbits, fruit trees, and various economic crops, and can frequently migrate between different host plants to establish populations [2,3,4]. However, with the widespread adoption of agroforestry planting patterns (e.g., intercropping and relay cropping under trees) in northern and northwestern China in recent years, the migration of *T. truncatus* across different ecological niches has intensified. Outbreaks have become frequent, and damage is increasingly severe on important economic woody fruit trees, such as jujube trees in Xinjiang [5]. The mites aggregate in large numbers on the undersides of jujube leaves, causing chlorosis, yellowing, and premature leaf drop, which severely weakens tree vigor and significantly reduces fruit quality and yield [6].

Herbivorous spider mites adjust their physiological and metabolic processes to acclimate to the nutritional components and chemical defenses of new host plants. In particular, the shift from herbaceous to woody hosts represents a major physiological transition and a challenge for ecological adaptation [7]. Compared with herbaceous plants, which have short life cycles, woody plants exhibit longer growth cycles and more complex tissue structures. In addition, woody plants generally contain richer and more systemic secondary defense metabolites, with potentially higher accumulated concentrations of certain classes such as phenolics and tannins [8,9]. Such differences in defense chemistry may pose greater detoxification challenges for spider mites feeding on woody plants than on herbaceous hosts [7,10]. Mechanistically, secondary metabolites from woody plants can interfere with herbivore performance by reducing digestive efficiency, generating oxidative stress, and disrupting cellular homeostasis. Phenolic compounds and tannins can bind dietary proteins and digestive enzymes, thereby limiting nutrient utilization, whereas terpenoid and other lipophilic compounds may require extensive metabolic transformation before elimination. To overcome these chemical barriers, spider mites rely on coordinated detoxification systems, including cytochrome P450 monooxygenases (P450s), glutathione S-transferases (GSTs), carboxylesterases (CarEs), and ATP-binding cassette (ABC) transporters, which participate in oxidation, conjugation, hydrolysis, and excretion of plant-derived toxic compounds [11,12]. Therefore, adaptation to woody hosts likely requires enhanced detoxification capacity and metabolic plasticity compared with feeding on herbaceous plants.

To adapt to the chemical defenses of host plants, herbivorous pests have evolved efficient detoxification metabolic systems. Among these, cytochrome P450 monooxygenases (CYP450), carboxylesterases (CarE), and glutathione S-transferases (GST) are key enzyme families involved in the metabolism of xenobiotic compounds [13,14]. Host plants can induce changes in the expression of detoxification enzyme-related genes, thereby enhancing the adaptability of pests to different hosts [15]. For example, genomic studies of the two-spotted spider mite (*Tetranychus urticae*) have revealed significant expansions of the CYP and GST gene families, which are closely associated with polyphagous adaptation [16]. Moreover, different host conditions can induce marked transcriptional responses of detoxification-related genes in spider mites [17,18]. In lepidopteran pests, host plant secondary metabolites can also significantly induce the expression of CarE and CYP450-related genes, thereby improving insect adaptation to host plants [19,20]. For instance, the gut-specific carboxylesterase gene SlCOE030 in the polyphagous pest Spodoptera litura was shown to be inducibly expressed after exposure to nicotine and cyantraniliprole, and its enhanced expression contributed to increased tolerance to these chemical stresses, highlighting the important role of specific CarE genes in insect detoxification and environmental adaptation. RNA interference (RNAi) technology provides an important tool for functional gene studies in pests and the development of green pest management strategies [21]. Previous studies have shown that targeted silencing of detoxification-related genes can affect pest survival, development, and reproduction [22,23]. Therefore, screening candidate functional genes from host-induced detoxification responses is of great significance for understanding pest adaptation processes and identifying potential RNAi targets.

To date, research on host adaptation in *Tetranychus truncatus* has focused mainly on herbaceous plants. Its adaptation mechanisms to woody hosts remain largely unknown. Common bean (*Phaseolus vulgaris*), cucumber (*Cucumis sativus*), and jujube (*Ziziphus jujuba*) are widely grown crops in northern China. In the field, intercropping, relay cropping, and rotation systems allow *T. truncatus* to move and transfer among these hosts. In particular, it is not yet clear which detoxification enzyme genes are specifically induced when *T. truncatus* shifts from herbaceous to woody hosts, or whether these genes directly participate in detoxifying the chemical defenses of woody plants. To address this, we transferred *T. truncatus* from common bean to cucumber and jujube. Mites were reared on each host for more than five generations. We then measured the activities of GST, CarE, and CYP450 enzymes, and analyzed the expression patterns of related genes using RT-qPCR. We also used RNA interference to explore the function of a key detoxification enzyme gene. This study aims to characterize the detoxification responses induced by different host plants. Our findings will provide a theoretical basis for understanding how spider mites adapt to woody plants and for screening RNAi targets.

## 2. Materials and Methods

### 2.1. Test Insects and Host Plants

The experimental *T. truncatus* mites were originally collected from a bean field in the suburbs of Lanzhou in 2020 and subsequently reared continuously on common bean (*P. vulgaris* 81-6 Didou, herbaceous) for more than four years in the Laboratory of Integrated Pest Management, College of Plant Protection, Gansu Agricultural University. Using common bean as the control host, the mites were transferred to cucumber (*C. sativus* Xinchun No. 4, herbaceous) and jujube (*Z. jujuba* Guoguang, woody) and reared for more than five consecutive generations on each host to establish different host-adapted populations. All populations were maintained in a climate chamber under conditions of 25 ± 1 °C, 60% ± 5% relative humidity, and a photoperiod of 16L:8D.

### 2.2. Determination of Detoxification Enzyme Activities

The common bean-reared population served as the control. *T. truncatus* populations continuously adapted to cucumber and jujube for five consecutive generations (F_5_) were selected for detoxification enzyme activity assays. For each treatment, 200 four-day-old adult female mites were collected into a 1.5 mL centrifuge tube, immediately frozen in liquid nitrogen, and stored at −80 °C until use. Three biological replicates were set up for each treatment. GST and CarE activities were determined using assay kits (Beijing Solarbio Science & Technology Co., Ltd., Beijing, China), and CYP450 content was measured using an ELISA kit (Shanghai Youxuan Biotechnology Co., Ltd., Shanghai, China), following the manufacturers’ protocols.

### 2.3. Transcriptome Data Analysis and Candidate Gene Screening

A total of 24 putative CarE genes were identified from the *T. truncatus* transcriptome database previously sequenced by our laboratory (submitted to the NCBI SRA database under accession numbers SRR21659835–SRR21959850). Transcriptome assembly sequences were subjected to redundancy removal and sequence validation using CodonCode Aligner (https://www.codoncode.com/aligner/index.htm, (accessed on 15 April 2026)) and DNAMAN (v9.0; Lymnon Bio-Soft, Quebec, QC, Canada). Homologous sequences were queried and screened using BLAST INPUT (Ghent University, Ghent, Belgium; http://bioinformatics.psb.ugent.be/blast/moderated/, (accessed on 16 April 2026)) to verify the accuracy of functional annotation. To further characterize the structural features of these CarE proteins, functional domains were predicted using the SMART software (European Molecular Biology Laboratory, Heidelberg, Germany; http://smart.embl-heidelberg.de/, (accessed on 16 April 2026)), and the isoelectric points (pI) and molecular weights of CarE genes were calculated using the ExPASy proteomics server (Swiss Institute of Bioinformatics; http://web.expasy.org/protparam/, (accessed on 16 April 2026)). Transmembrane domains were localized using TMHMM (version 5.5.9; http://www.cbs.dtu.dk/services/TMHMM/, (accessed on 16 April 2026)). Motif annotation and visualization were performed using the MEME online motif discovery tool (version 5.5.9; http://meme-suite.org/tools/meme, (accessed on 16 April 2026)). Finally, based on the sequences of the 24 CarE genes, a phylogenetic tree was constructed using the neighbor-joining method implemented in Molecular Evolutionary Genetics Analysis (MEGA) version 7.0 to elucidate the evolutionary relationships and functional divergence of these genes.

### 2.4. RNA Extraction and RT-qPCR Analysis

Total RNA was extracted using TRIzol reagent (Invitrogen, Carlsbad, CA, USA). Approximately 200 adult female mites per sample were used, with three biological replicates. RNA concentration and purity were assessed using a NanoPhotometer-N50 (Implen GmbH, Munich, Germany). Qualified RNA was reverse-transcribed using the PrimeScript RT reagent kit (TaKaRa, Dalian, China) to synthesize cDNA, which was stored at −20 °C until use. RT-qPCR was performed using the SYBR Green I method (NS SYBR Plus kit, Nearshore Protein Technology Co., Ltd., Shanghai, China) on a real-time quantitative PCR system (Applied Biosystems, Waltham, MA, USA). The reaction mixture (10 µL) contained cDNA, primers, and fluorescent dye. The thermal cycling protocol was as follows: 95 °C for 2 min, followed by 40 cycles of 95 °C for 15 s and 60 °C for 30 s. Actin and RPS18 were used as reference genes. Relative expression levels were calculated using the 2^−ΔΔCT^ method based on three technical replicates per sample.

### 2.5. dsRNA Synthesis and RNA Interference (RNAi)

Based on the RT-qPCR results, a detoxification-related gene that was significantly upregulated in jujube-fed mites was selected for RNAi analysis. The target gene, *TtCarE-4*, was identified from *T. truncatus*. Specific primers for dsRNA synthesis of the target gene and the negative control GFP gene were designed using NCBI Primer-BLAST, and the T7 promoter sequence (TAATACGACTCACTATAGGG) was added to the 5′ end of each primer. All primers were synthesized by Shanghai Qingke Biotechnology Co., Ltd., Shanghai, China after specificity verification. The primer sequences used for RT-qPCR and dsRNA synthesis are provided in Supplementary Tables S1 and S3.

A TaKaRa Taq™ kit (TaKaRa, Dalian, China) was used for PCR amplification using *T. truncatus* cDNA as a template. The reaction mixture (25 μL) contained 12.5 μL of 2× Taq Master Mix, 1 μL of cDNA template, 1 μL each of forward and reverse primers, and 9.5 μL of ddH_2_O. The PCR program was as follows: initial denaturation at 95 °C for 10 min; 38 cycles of denaturation at 95 °C for 30 s, annealing at 60 °C for 30 s, and extension at 72 °C for 30 s; and final extension at 72 °C for 10 min. The amplification products were detected by 1% agarose gel electrophoresis.

The PCR products were recovered and purified using a universal DNA purification kit (Shanghai Yuanye Bio-Technology Co., Ltd., Shanghai, China). First, the adsorption column was equilibrated; then an equal volume of binding solution PC was added to the PCR product, mixed thoroughly, and transferred to the adsorption column to adsorb DNA. After washing with PW rinse twice, the empty column was centrifuged to remove residual ethanol, and EB buffer was added to elute the DNA; the purified PCR product was finally obtained. The dsRNA was synthesized using the TranscriptAid T7 Kit (Beyotime Biotechnology, Shanghai, China). The reaction system included 5× Reaction Buffer, ATP, UTP, GTP, CTP, DNA template and Enzyme Mix, with a total volume of 20 μL. The mixture was incubated at 37 °C for 6 h to complete the transcription reaction.

After dsRNA was synthesized, DNase I was added to remove the template DNA, and then purified using phenol/chloroform extraction and ethanol precipitation. Specific steps included adding DNase I to digest the template DNA, terminating the reaction with EDTA; then adding enzyme-free water, sodium acetate, water-saturated phenol/chloroform mixture and chloroform in sequence, collecting the supernatant after low-temperature centrifugation; then adding absolute ethanol to precipitate dsRNA at −20 °C. The precipitate was collected by centrifugation, washed with 75% ice-cold ethanol, dried at room temperature, and resuspended in nuclease-free water. The dsRNA was dissolved in enzyme-free water, and a micro-volume spectrophotometer was used to detect its concentration and purity. When the OD260/OD280 value was between 1.8 and 2.0, the purity of dsRNA was considered to meet the requirements of subsequent tests and stored at −80 °C for future use.

RNA interference was conducted using an artificial feeding method. A dsRNA feeding device was constructed by referring to the existing leaf delivery method: four layers of paraffin film were laid on the bottom of an inverted Petri dish, a 100-mesh nylon mesh was placed on top, and 60 μL of *dsTtCarE-4*, *dsGFP* and sterile water treatment solution with a concentration of 1000 ng/μL were added, with *dsGFP* and water serving as controls. Then, the stretched sealing film was covered to form a sandwich feeding structure, and the device was placed in a Petri dish containing sponge to moisturize. Additionally, moist lens paper was used to surround the feeding area to prevent spider mites from escaping.

Each treatment included three biological replicates, and for each replicate, 300 four-day-old adult female mites were collected and fed continuously in darkness at 25 ± 1 °C for 24 h. Immediately after the 24 h feeding period, surviving mites from each replicate were divided into two groups: 50 randomly selected live mites were transferred to fresh jujube leaf discs for host fitness (biological effect) assessment, and the remaining live mites (approximately 200 per replicate) were collected, flash-frozen in liquid nitrogen, and stored at −80 °C for subsequent total RNA extraction and silencing efficiency evaluation.

### 2.6. Silencing Efficiency Assay

Total RNA was extracted from the spider mite samples (three biological replicates per treatment) that had been collected and frozen after feeding on treatment solutions under dark conditions for 24 h. After cDNA was synthesized, RT-qPCR was used to detect the relative expression of the target gene and calculate the silencing efficiency of the target gene. Silencing efficiency (%) = (1 − relative expression level of *dsTtCarE-4*-treated mites/relative expression level of *dsGFP*-treated control mites) × 100%.

### 2.7. Assessment of Biological Effects

Adult female mites that had fed on *dsTtCarE-4* under dark conditions for 24 h were transferred to jujube leaf discs. The experiment was conducted with three replicates, each consisting of 50 adult female mites. Mites were considered dead when no movement was observed upon gentle probing with a fine brush tip, and all four pairs of legs remained curled against the body. Mortality and oviposition were recorded every 24 h, and the cumulative mortality and oviposition rate were calculated over a 5-day period.

### 2.8. Analysis of dsRNA Conservation and Off-Target Risk

On-target and off-target analyses were conducted using an online website (College of Plant Protection, Southwest University, Chongqing, China; https://dsrna-engineer.cn/, (accessed on 20 April 2026)). Based on RNAi, the target effects of *dsTtCarE-4* on the harmful spider mite were evaluated. Off-target effects on non-harmful organisms such as *Neoseiulus barkeri*, *Harmonia axyridis*, *P. vulgaris*, *C. sativus*, and *Z. jujuba* were also analyzed to ensure the safety of dsRNA on non-target organisms.

### 2.9. Data Analysis

Statistical analysis and graphing were performed using GraphPad Prism 9.0. All data were assessed for normality by the Shapiro–Wilk test and analyzed for homogeneity of variances by Levene’s test before performing parametric tests. For data that conformed to a normal distribution and had homogeneous variances (such as enzyme activity, egg-laying volume, and relative gene expression), one-way ANOVA was used, followed by Tukey’s HSD method for multiple comparison tests (*p* < 0.05). For percentage data (mortality rates), based on transformed data analysis, an arcsine square root transformation was performed prior to the analysis of variance to satisfy the assumption of homogeneity of variances. If data did not conform to a normal distribution or displayed heteroscedasticity, the non-parametric Kruskal-Wallis test was used. Data are expressed as “mean ± standard error (SE)”.

## 3. Results

### 3.1. Changes in GST and CarE Enzyme Activities, and CYP450 Content in T. truncatus Adult Female Mites on Different Host Plants

**Figure 1:**
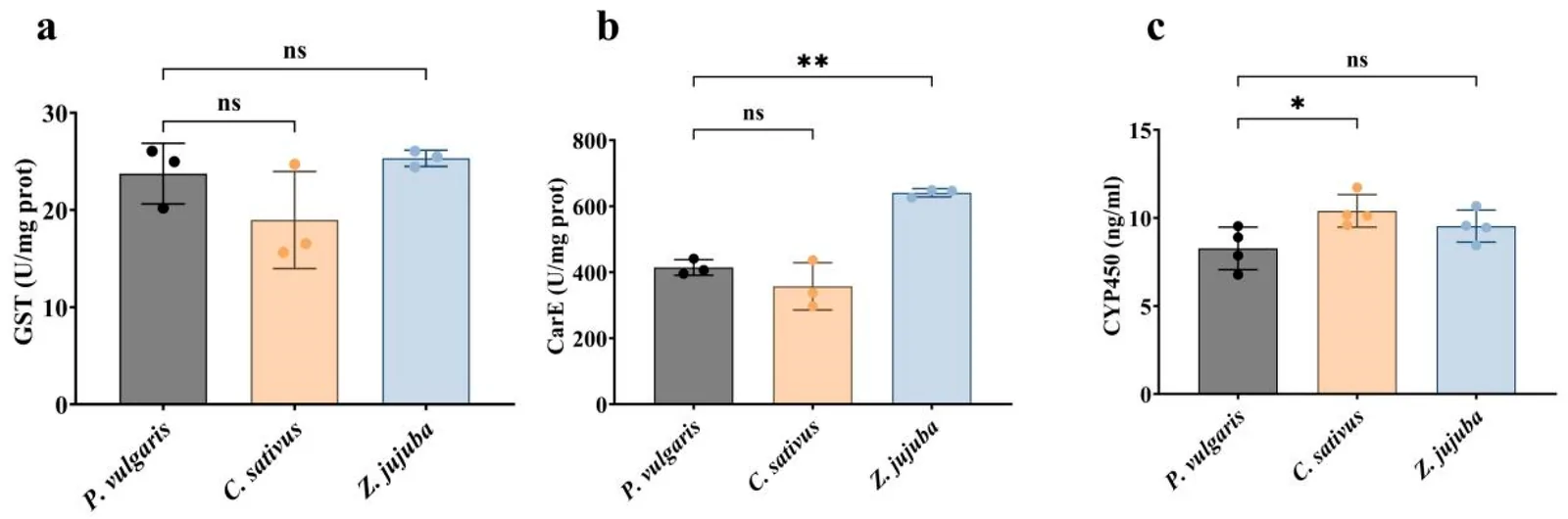
Effects of different host plants on detoxification enzyme activities and relative content in *T. truncatus.* (**a**) Glutathione S-transferase (GST) activity; (**b**) carboxylesterase (CarE) activity; (**c**) relative cytochrome P450 (CYP450) content in adult female mites continuously reared on common bean (*P. vulgaris*), cucumber (*C. sativus*), and jujube (*Z. jujuba*) for more than five generations. Bars represent mean ± SE (*n* = 3). Asterisks indicate significant differences among treatments (* *p* < 0.05, ** *p* < 0.01), and ‘ns’ indicates no significant difference.

Different host plants had varying effects on the detoxification enzyme activities of *T. truncatus* (Figure 1). GST activity showed no significant difference between the cucumber and jujube treatment groups (*p* > 0.05). CarE activity was significantly affected by host plant species (*p* < 0.05), with CarE activity being significantly higher in mites fed on the woody host jujube than in those fed on common bean and cucumber. The CarE activity in the jujube-fed mites was 1.54-fold and 1.79-fold higher than that in the common bean and cucumber-fed mites, respectively, while no significant difference was observed between the common bean and cucumber-fed mites. CYP450 content also differed significantly among host plants (*p* < 0.05). Mites fed on the herbaceous host cucumber exhibited significantly higher CYP450 content than those fed on common bean, with a 1.26-fold increase, whereas no significant difference was detected between the jujube-fed mites and the other two treatments. These results indicated that host plants significantly affected CarE and CYP450 content in *T. truncatus*: the woody host (jujube) primarily induced CarE, while the herbaceous host (cucumber) primarily induced CYP450 content. In contrast, GST activity was less sensitive to host plant variation. CarE may play an important role in the adaptation of *Tetranychus truncatus* to the woody host plant jujube.

### 3.2. Identification and Sequence Characterization of the CarE Gene Family in T. truncatus

Based on the transcriptome data of *T. truncatus*, a total of 24 carboxylesterase family genes were identified and sequentially named *TtCarE-1* to *TtCarE-24*. Physicochemical properties are shown in Supplementary Table S2. The amino acid sequence lengths of these 24 TtCarE proteins range from 261 aa (*TtCarE-24*) to 1072 aa (*TtCarE-2*), and the open reading frame (ORF) lengths range from 783 bp to 3216 bp. The relative molecular masses of the proteins range from 30.05 kDa to 118.59 kDa, and the theoretical isoelectric points (pI) ranged from 4.98 (*TtCarE-14*) to 8.62 (*TtCarE-9*), with most exhibiting weakly acidic properties. Furthermore, signal peptide and transmembrane domain prediction results indicated that *TtCarE-3*, *TtCarE-11*, *TtCarE-13*, *TtCarE-14*, *TtCarE-16*, *TtCarE-19*, *TtCarE-20*, and *TtCarE-21* contain signal peptide sequences, while *TtCarE-1*, *TtCarE-2*, *TtCarE-3*, *TtCarE-4*, *TtCarE-5*, *TtCarE-10*, and *TtCarE-17* possess one transmembrane domain. These results suggest that some TtCarE proteins may be membrane-bound or participate in metabolic reactions as secreted proteins.

**Figure 2:**
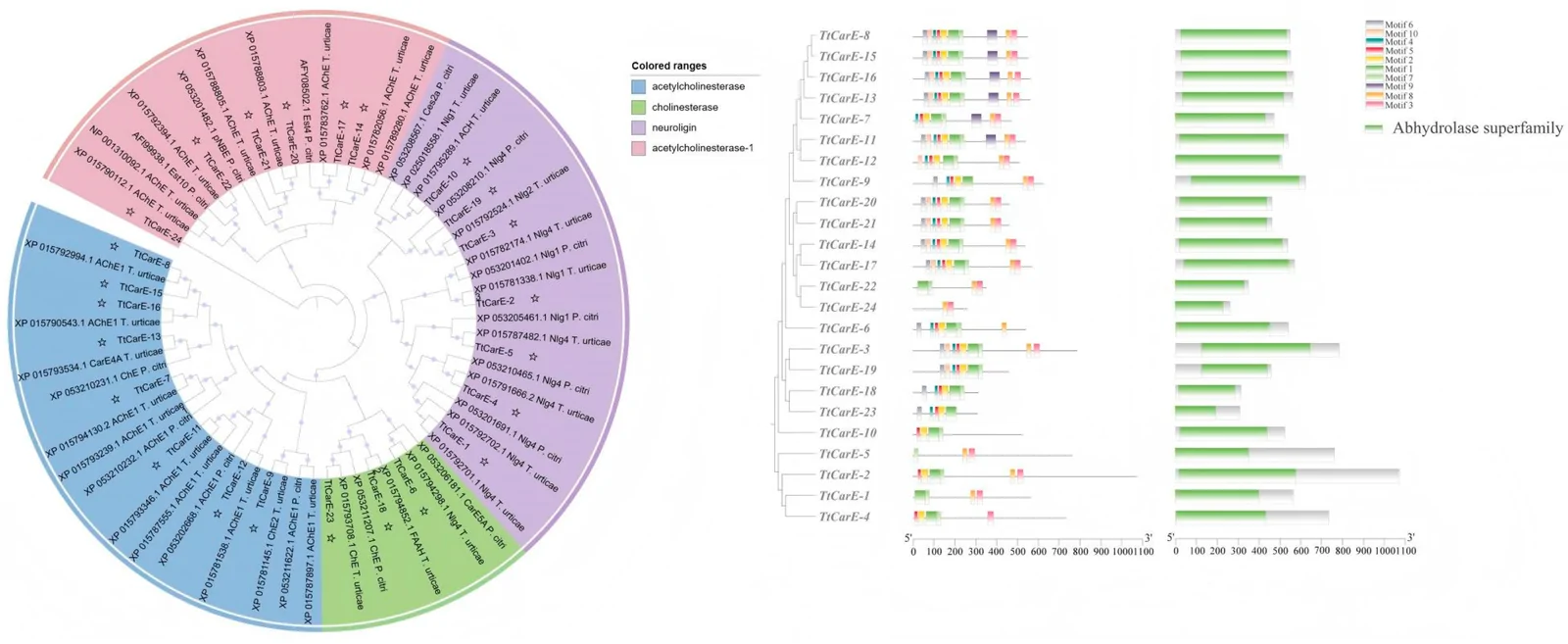
Phylogenetic relationships and conserved motif composition of carboxylesterase family proteins from *T. truncatus*, *T. urticae*, and *P. citri*. Conserved motifs are shown on the right. Members were classified into major carboxylesterase subfamilies according to their phylogenetic relationships. *T. truncatus* carboxylesterases (TtCarEs) are marked with a star in the corresponding clades. The *T. truncatus* CarE sequences were identified from transcriptomic datasets deposited in the NCBI Sequence Read Archive (SRA) under accession numbers SRR21659835–SRR21959850; the accession numbers of the reference CarE sequences from *T. urticae* and *P. citri* are provided in the phylogenetic tree. Stars indicate the 24 carboxylesterase genes identified in *T. truncatus*. Purple dots indicate bootstrap support values at the corresponding nodes.

To examine evolutionary relationships, we constructed a phylogenetic tree using CarE protein sequences from *T. truncatus*, *T. urticae*, and *Panonychus citri* (Figure 2). All CarE members fell into four major clades: neuroligin (NLGN), acetylcholinesterase (AChE), cholinesterase (ChE), and AChE1. The 24 TtCarE proteins were distributed across all four clades, most clustering closely with their *T. urticae* homologs, indicating strong evolutionary conservation. Notably, several TtCarE members formed distinct sub-branches (e.g., *TtCarE-4*, *TtCarE-8*, *TtCarE-15*), hinting at possible functional divergence.

Further conserved domain and motif pattern analysis (Figure 2) confirmed that all 24 *TtCarE* protein sequences contain the typical α/β hydrolase fold superfamily (Abhydrolase superfamily) conserved domain, which is the core feature responsible for the catalytic function of the carboxylesterase family. Moreover, TtCarE members that clustered in the same branch of the phylogenetic tree with close evolutionary relationships often shared highly similar motif compositions and arrangements (e.g., *TtCarE-8*, *TtCarE-15*, *TtCarE-16*, and *TtCarE-13* exhibited nearly identical motif patterns). This strong consistency between protein conserved structure and phylogenetic relationships further validates the accuracy and reliability of the classification of the CarE gene family.

### 3.3. Changes in Carboxylesterase Gene Expression Levels in T. truncatus on Three Host Plant Species

**Figure 3:**
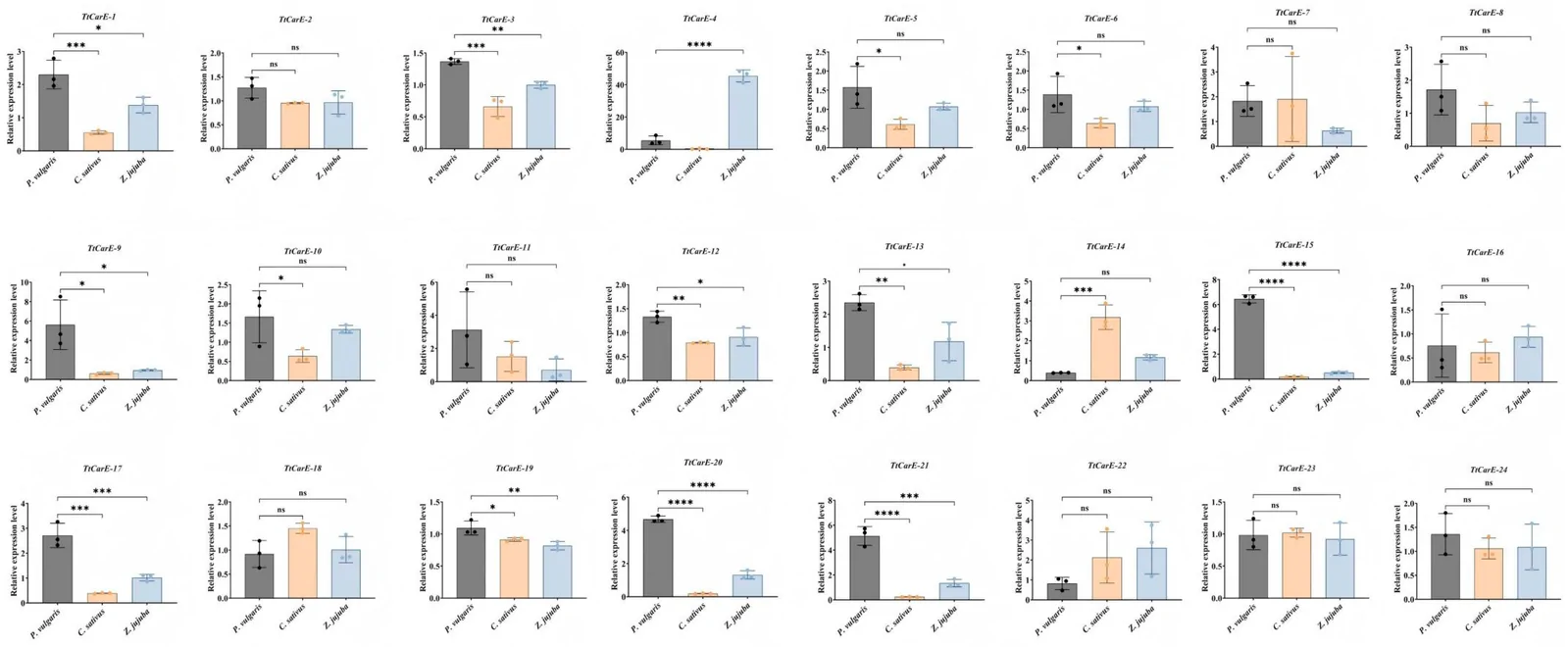
Relative expression levels of *TtCarE* gene family members in *T. truncatus* after transfer to different host plants (common bean, cucumber, and jujube). Bars represent mean ± SE (*n* = 3). Asterisks indicate significant differences among treatments (* *p* < 0.05, ** *p* < 0.01, *** *p* < 0.001, **** *p* < 0.0001), and ‘ns’ indicates no significant difference.

RT-qPCR results showed that different host plant species significantly influenced the expression patterns of *TtCarE* genes (Figure 3). Most *TtCarE* genes (such as *TtCarE-1*, *TtCarE-3*, *TtCarE-5*, *TtCarE-9*, *TtCarE-13*, *TtCarE-15*, *TtCarE-17*, *TtCarE-20*, and *TtCarE-21*) were highly expressed in the original host, common bean, but their expression levels generally decreased after transfer to cucumber or jujube (Figure 3).

In contrast, *TtCarE-4* showed a significant and specific upregulation in response to jujube, with expression levels 8-fold and 160-fold higher than those on common bean and cucumber, respectively (*p* < 0.05). This gene exhibited the most pronounced response to jujube among all TtCarE members, and its expression trend was generally consistent with changes in CarE enzyme activity, suggesting that *TtCarE-4* may play an important role in the adaptation of *T. truncatus* to jujube.

Furthermore, *TtCarE-14* showed significantly higher expression on cucumber than on common bean or jujube (*p* < 0.05). No significant differences among host plants were observed for *TtCarE-2, TtCarE-11, TtCarE-16, TtCarE-23, and TtCarE-24*.

### 3.4. Detection of TtCarE-4 Gene Silencing Efficiency

**Figure 4:**
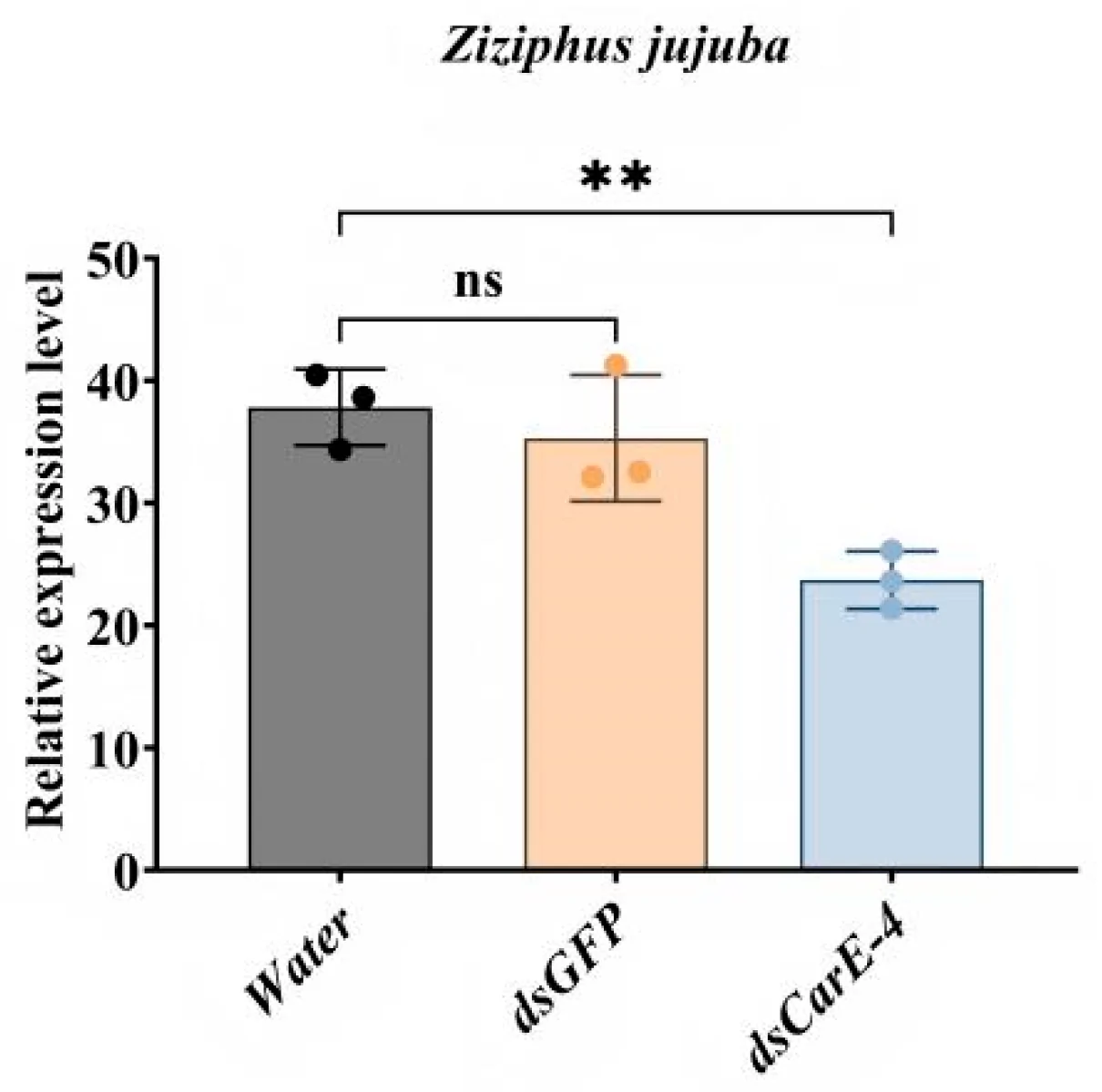
Silencing efficiency of the *TtCarE-4* gene in *T. truncatus* following dsRNA feeding. Relative expression of *TtCarE-4* at 24 h after feeding on *dsTtCarE-4*. Sterile water and *dsGFP* treatments served as controls. Bars represent mean ± SE (*n* = 3). Asterisks indicate significant differences among treatments (** *p* < 0.01), and ‘ns’ indicates no significant difference.

Based on the aforementioned gene expression analyses, *TtCarE-4*, which exhibits high differential expression levels on jujube trees, was selected as target. After 24 h of RNA interference, the expression level of the target gene significantly decreased after *dsTtCarE-4* treatment (Figure 4). Compared with the water and dsGFP control groups, the expression level of *TtCarE-4* decreased by approximately 37.3% and 32.8%, respectively (*p* < 0.05). There was no significant difference between the water group and the dsGFP group (*p* > 0.05).

### 3.5. Effects of Silencing the TtCarE-4 Gene on the Mortality and Fecundity of T. truncatus

**Figure 5:**
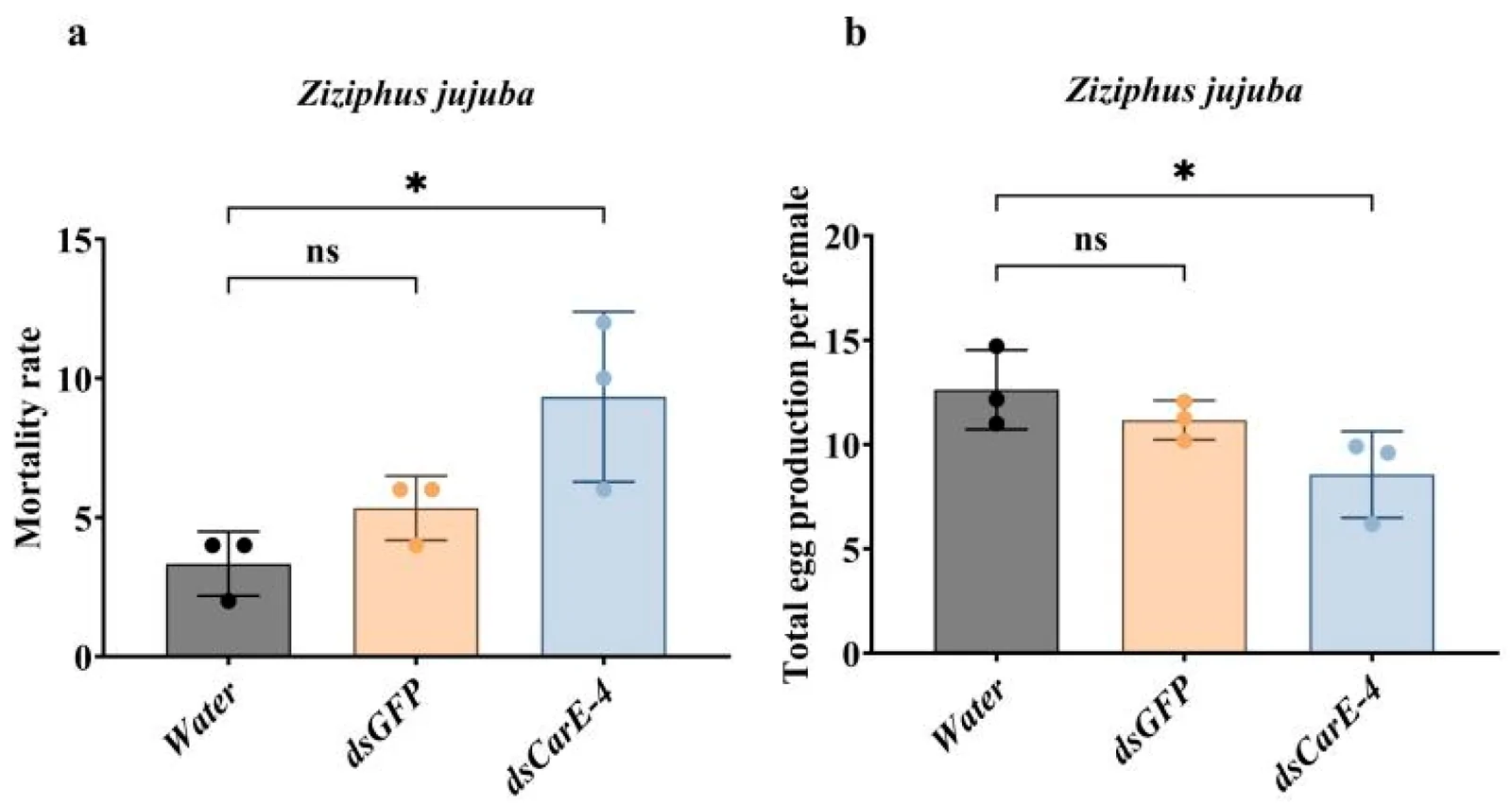
Functional validation of *TtCarE-4* by RNA interference in *T. truncatus*. (**a**) Cumulative mortality of adult female mites and (**b**) total fecundity per female following *TtCarE-4* silencing. Bars represent mean ± SE (*n* = 3). Asterisks indicate significant differences among treatments (* *p* < 0.05), and ‘ns’ indicates no significant difference.

After being transferred to jujube tree leaves for continued rearing, the mortality rate in the *dsTtCarE-4* treatment group was significantly increased (Figure 5). Compared with the water and dsGFP control groups, the mortality rate of spider mites in the *dsTtCarE-4* treatment group reached approximately 9.2%, while the mortality rates in the water and *dsGFP* groups were only approximately 3.3% and 5.3%, respectively (*p* < 0.05), and there was no significant difference between the water and *dsGFP* groups. Silencing *TtCarE-4* significantly reduced the oviposition rate of *T. truncatus* (Figure 5). Compared with the clean water control group, the oviposition rate per female in the *dsTtCarE-4* treatment group was 9.6 eggs per female, which was significantly lower than that in the water control group (approximately 12.6) and the *dsGFP* control group (approximately 11.2) (*p* < 0.05). There was no significant difference between the *dsGFP* and clean water control groups.

### 3.6. Target Conservation and Off-Target Risk Analysis of TtCarE-4 dsRNA

In this study, the gene sequence was compared with the nucleic acid sequences of target organisms (*T. truncatus*) and non-target organisms (*N. barkeri*, *H. axyridis*, *P. vulgaris*, *C. sativus*, and *Z. jujuba*) to detect the potential off-target counts of the endogenous gene *TtCarE-4* in the genomes of both target and non-target organisms. The results showed that among target organisms, *T. truncatus* had the highest number of off-target genes, reaching 291 times; while among non-target organisms, *H. axyridis* had the lowest number, with three predicted off-target matches. (Table 1), suggesting that this gene is a safe target for RNAi.

## 4. Discussion

The host shift of *T. truncatus* from herbaceous to woody plants is not an isolated phenomenon observed solely in laboratory settings. In arid and semi-arid fruit-producing regions of northern China, orchard intercropping (e.g., planting legumes or cucurbit vegetables under jujube trees) is widely used to improve land use efficiency and conserve soil moisture. This extensive spatial niche overlap creates conditions for spider mites to migrate from senescing herbaceous ground cover to upper canopy woody hosts like jujube trees during seasonal transitions [24,25]. During the coevolution of plants and herbivorous insects (mites), host plants impose continuous selective pressure on feeders through diverse secondary metabolites. In response, herbivorous pests (mites) adapt to different hosts by regulating their detoxification metabolic systems [14,17,26]. The detoxification enzyme systems in herbivorous insects primarily include CYP450, GST, and CarE, all of which play core metabolic roles in response to plant secondary metabolites and xenobiotic stress [20,27,28]. Therefore, elucidating the specific detoxification adaptation mechanisms of *T. truncatus* to woody hosts not only complements theories on the coevolution of polyphagous pest mites but also holds practical significance for integrated pest management (IPM) strategies within such complex agricultural ecosystems.

We found that detoxification enzyme responses of *T. truncatus* differed markedly between herbaceous hosts (common bean and cucumber) and the woody host jujube. Different host plants significantly affected CarE activities and CYP450 content, but the induction patterns were distinct: the woody host (jujube) specifically induced elevated CarE activity, whereas the herbaceous host (cucumber) specifically induced elevated CYP450 activity. GST activity did not differ significantly among hosts. These findings suggest that chemical defenses of different host plants may activate distinct detoxification pathways in a targeted manner. Although our results show that cucumber significantly induces CYP450 activity, *T. truncatus*, as a typical polyphagous pest mite, commonly shifts among herbaceous hosts, and the P450-mediated adaptation mechanisms involved have been extensively documented [7,17]. In contrast, the transition from herbaceous to woody plants (jujube) represents a more dramatic ecological niche shift and challenge from secondary metabolites [29]. CarE is recognized as an important detoxification enzyme enabling herbivorous insects to adapt to different host plants. Previous studies have shown that in Helicoverpa armigera and *T. urticae*, esterase genes are rapidly upregulated to enhance xenobiotic metabolism upon host transfer [6,30]. Consistent with these findings, CarE activity was significantly elevated in *T. truncatus* feeding on jujube, suggesting a central role for carboxylesterases in facilitating this herbaceous-to-woody host transition. Studies have also found that host plants can not only influence the growth and development of phytophagous pests, but may also affect their sensitivity to pesticides by inducing detoxification metabolic systems [17]. For example, in *Nilaparvata lugens*, the Akt-FoxO signaling pathway coordinately regulates the expression of multiple P450 genes, enabling simultaneous adaptation to rice allelochemicals and insecticides [31]. In the present study, cucumber significantly induced CYP450 activity, whereas jujube enhanced CarE activity, indicating that *T. truncatus* may exhibit differential detoxification responses depending on the host plant context. Host-induced detoxification responses may also alter pesticide susceptibility in mite populations [32]. Therefore, in multi-host cropping systems or under field rotation conditions, attention should be paid to the differential induction of detoxification metabolism in pest mites and the potential driving role in resistance evolution.

At the gene expression level, different host plants also exerted varying effects on the TtCarE gene family. RT-qPCR results showed that the expression level of *TtCarE-4* in jujube was significantly higher than in common bean and cucumber, and this trend was generally consistent with changes in CarE enzyme activity. Previous studies have indicated that CarE is not only involved in the metabolism of plant secondary metabolites but also closely related to insect environmental adaptation and metabolic resistance formation [10,28]. Genome analysis of *T. truncatus* showed that compared with the generalized two-spotted spider mite (*T. urticae*), *T. truncatus* has fewer detoxification-related genes, but its extensive interpopulation transcriptional variation may be an important strategy for adapting to low-quality host plants [2]. In this study, the host-specific expression variation of *TtCarE-4* may also play a particular role in the adaptation of *T. truncatus* to the chemical environment of specific hosts. Nevertheless, as further validation using metabolic substrates or enzyme activity changes is lacking, the precise mechanism of action requires additional investigation.

RNA interference results further demonstrated that silencing *TtCarE-4* adversely affected the mortality rate of *T. truncatus* and significantly reduced the total number of eggs laid per female. Similar findings have been reported in *Hyphantria cunea* and *Acyrthosiphon pisum*. In H. *cunea*, the carboxylesterase gene *HcCarE6* exhibited the highest expression levels on less preferred host plants, and silencing of this gene significantly reduced fitness, as evidenced by reduced larval weight and increased mortality; conversely, overexpression of *HcCarE6* in transgenic Drosophila significantly enhanced tolerance to the plant defense compounds coumarin and cytisine [33]. In *A. pisum*, *ApCarE4* expression was significantly upregulated after feeding on resistant alfalfa varieties, whereas RNA interference of this gene led to a significant increase in mortality [34]. These findings are highly consistent with the results of the present experiment, in which *TtCarE-4* showed high expression on a specific host, and silencing resulted in adverse phenotypes, suggesting that the CarE family plays a similar and critical role in host adaptation across multiple polyphagous pests. In addition to their established role in detoxification, some carboxylesterases have been shown to participate in physiological processes associated with development and reproduction. RNAi-mediated suppression of *EST-1* in *Sogatella furcifera* significantly reduced egg production, ovarian protein accumulation, and adult longevity, whereas silencing of a juvenile hormone esterase-related gene affected developmental programming in *Sesamia nonagrioides* [35,36]. Previous studies have indicated that some detoxification genes, in addition to participating in xenobiotic metabolism, may also be involved in insect growth, development, energy allocation, and reproductive processes [21,37,38]. It should be noted that RNAi efficiency in spider mites is often moderate, influenced by factors such as target gene, dsRNA concentration, developmental stage, and mite species [39,40,41]. While *TtCarE-4* silencing efficiency was moderate (approximately 37% reduction in transcript levels), RNAi treatment significantly increased mortality and reduced female fecundity, indicating that partial suppression of *TtCarE-4* is sufficient to impair the fitness of *T. truncatus*. Similar observations have been reported in spider mites, where moderate transcript reduction can still result in pronounced biological effects despite relatively limited RNAi efficiency. Therefore, the observed phenotypic changes strongly support an important role of *TtCarE-4* in host adaptation.

RNA interference (RNAi) efficiency in spider mites is influenced by multiple biological and technical factors. Exogenously supplied double-stranded RNA (dsRNA) is readily degraded by environmental nucleases as well as by nucleases present in the gut lumen and hemolymph, reducing the amount of intact dsRNA available for gene silencing. In addition, orally delivered dsRNA generally exhibits limited systemic spread and primarily affects gut tissues, thereby restricting the silencing of genes expressed in other tissues. Considerable inter-individual variation in RNAi responsiveness further limits overall knockdown efficiency, with only a proportion of treated mites exhibiting detectable transcript suppression. Moreover, RNAi efficiency varies substantially among target genes, ranging from approximately 23% to over 70% under similar experimental conditions, and the degree of transcript reduction is not always positively correlated with phenotypic severity. Functional redundancy within detoxification enzyme families, including carboxylesterases (CarEs), glutathione S-transferases (GSTs), and cytochrome P450 monooxygenases (CYP450s), may further compensate for the loss of individual genes, thereby masking the effects of single-gene silencing [42,43].

Recent studies have proposed several strategies to improve RNAi efficiency in spider mites. Optimizing dsRNA delivery methods, particularly the egg-soaking approach, has proven to be an effective, practical, and cost-efficient strategy for gene silencing. Nanoparticle-mediated delivery systems, such as single-walled carbon nanotubes, can enhance dsRNA stability, facilitate cellular uptake, and improve tissue-specific accumulation. Furthermore, fusion dsRNA constructs targeting multiple genes within the same biological pathway have been shown to achieve stronger and more coordinated gene silencing than single-gene dsRNA. Optimizing dsRNA length and selecting genes that are highly expressed in gut tissues or encode membrane transport proteins and components of the 26S proteasome pathway have also been reported to enhance environmental RNAi responses.

Taken together, although the silencing efficiency of *TtCarE-4* was moderate in the present study, the significant increases in mortality and reductions in female fecundity demonstrate that even partial suppression of this gene is sufficient to compromise the fitness of *T. truncatus*. These findings support the involvement of *TtCarE-4* in adaptation to woody host plants. Future studies integrating improved RNAi delivery strategies with carboxylesterase activity assays and multi-gene functional analyses will provide a more comprehensive understanding of the molecular mechanisms underlying *TtCarE-4*-mediated host adaptation and facilitate the development of RNAi-based management strategies for spider mites [44]. In this study, we conducted a bioinformatic analysis to evaluate the potential off-target effects of dsRNA targeting *TtCarE-4* using available genome resources of non-target organisms. The results revealed no significant sequence similarity between the designed dsRNA region and the examined non-target genomes, suggesting a high predicted specificity toward *T. truncatus*. However, these findings represent only a preliminary assessment based on sequence-level prediction and cannot completely exclude potential non-target effects under practical application conditions. Therefore, further biological validation, including direct feeding assays using representative beneficial arthropods such as *Harmonia axyridis*, is necessary to evaluate possible effects on survival, development, and reproduction. Such assessments will be critical for comprehensive environmental risk evaluation and for determining the practical feasibility of developing *TtCarE-4*-based RNAi strategies for sustainable pest management [45].

In addition to dsRNA delivery and cellular uptake, the stability and intracellular processing of exogenous dsRNA may also contribute to the variable RNAi efficiency observed in spider mites. dsRNases, which degrade exogenous dsRNA, have been proposed as potential barriers to environmental RNAi in several insect taxa [46]. However, compared with Lepidoptera, Coleoptera, and Orthoptera, information on dsRNase-mediated dsRNA degradation in Tetranychidae remains limited, and direct evidence for the contribution of dsRNases to RNAi efficiency in *T. truncatus* is currently lacking. Studies in the closely related two-spotted spider mite, *Tetranychus urticae*, have shown that dsRNA processing and stability can influence RNAi efficiency. In particular, longer dsRNAs (≥400 bp) were more efficiently processed and induced stronger RNAi responses than shorter dsRNAs, suggesting that dsRNA processing within mites may substantially affect RNAi outcomes [47]. Moreover, RNAi efficiency varies among target genes and delivery methods in *T. urticae*, further indicating that dsRNA uptake, stability, and intracellular processing may contribute to differences in RNAi performance [47,48]. Therefore, the moderate knockdown efficiency of *TtCarE-4* observed in the present study may partly reflect limitations associated with dsRNA stability and processing, although the specific contribution of dsRNases in *T. truncatus* remains to be experimentally determined. Future studies investigating the presence, expression, and functional roles of candidate dsRNases in *T. truncatus* and related tetranychid mites would help clarify their contribution to RNAi efficiency.

In summary, different host plants induce distinct detoxification responses in *T. truncatus*. During the transition from herbaceous to woody (jujube) hosts, the carboxylesterase system shows a specific surge. Silencing *TtCarE-4* significantly reduces mite fitness on jujube. It remains unclear whether this fitness cost results solely from impaired detoxification of jujube-specific metabolites or also involves disruption of fundamental physiology. Notably, *TtCarE-4* expression is very low on the original common bean host but increases eightfold after transfer to jujube, suggesting an inducible role primarily in woody plant chemical defense rather than as a housekeeping gene. Moreover, our findings primarily reflect the stabilized response after host adaptation rather than the dynamic molecular changes occurring during the initial host transition. Therefore, future research should focus on elucidating the molecular mechanisms that govern this dynamic transition phase. In parallel, RNAi fitness assays performed in the original host, common bean, will help to distinguish between the potential physiological roles of TtCarE-4 in basic development and its specific contributions to host adaptation.

## 5. Conclusions

Different host plants induced distinct detoxification responses in *Tetranychus truncatus*. The woody host Ziziphus jujuba specifically induced CarE activity and markedly upregulated *TtCarE-4* expression. RNAi-mediated silencing of *TtCarE-4* significantly increased mite mortality and reduced fecundity, indicating that this gene contributes to adaptation to woody hosts and represents a promising candidate target for RNAi-based spider mite management.

## Figures and Tables

**Table 1:** On-target analysis of *TtCarE-4* in three target organisms and non-target organisms.

Conservativeness Analysis					Security Analysis				
Species	Total In-Target Times	Top 10 Genes			Species	Total In-Target Times	Top 10 Genes		
		Species	Gene ID	In-Target Times			Species	Gene ID	Off-Target Times
*T. truncatus*	291	*T.* *truncatus*	TRINITY_DN9069_c0_g1_i2	291	*P. vulgaris*	3	*P. vulgaris*	XM_007152988.1	3
*T. urticae*	176	*T. urticae*	XM_015936180.2	171	*C. sativus*	4	*C. sativus*	XM_004140466.3	4
*P. ulmi*	11	*P. ulmi*	TRINITY_DN14669_c0_g1_i1	11	*Z. jujuba*	3	*Z. jujuba*	XM_048462961.2	3
		*T. truncatus*	TRINITY_DN9069_c0_g1_i1	139	*N. barkeri*	3	*N. barkeri*	gene-LOC100902902	3
		*P. ulmi*	TRINITY _DN390 _c0_g2_i1	5	*H. axyridis*	3	*H. axyridis*	XM_045623417.1	3
							*P. vulgaris*	XM_007152989.1	3
							*C. sativus*	XR_969289.2	2
							*Z. jujuba*	XM_060814733.1	2
							*H. axyridis*	XM_045614553.1	3

## Data Availability

The transcriptome datasets generated and analyzed during this study have been deposited in the NCBI Sequence Read Archive (SRA) under accession numbers SRR21659835–SRR21959850. Other data supporting the findings of this study are available from the corresponding author upon reasonable request.

## Supplementary Materials

The following supporting information can be downloaded at: https://www.mdpi.com/article/10.3390/insects17090913/s1, Table S1: Primers used for RT-qPCR and dsRNA synthesis; Table S2: Physicochemical characteristics of the 24 identified TtCarE proteins; Table S3: Predicted off-target analysis of *dsTtCarE-4* in target and non-target organisms. Figure S1: Experimental setup of the oral dsRNA feeding assay for *T. truncatus*.
