# Supplementary material for: Host-Specific Induction and Functional Characterization of the Carboxylesterase TtCarE-4 in Tetranychus truncatus Adaptation to Woody Host Plants
Source: Insects. 2026 Sep 1;17(9):913. doi: 10.3390/insects17090913 (PMC13607576; doi:10.3390/insects17090913)
Supplement: Supplementary file 1 [file insects-17-00913-s001.zip › insects-4470710-supplementary.pdf]

Table S1 Primers used for RT-qPCR and dsRNA synthesis.

| Gene name | Gene ID | Primer sequence (5’-3’) |
|---|---|---|
| *TtCarE-1* | Isoform0002199 | F:CCGATGGGCAGAGGACTTTT<br>R:CAGAGTTAGAGGGTCACCTGG |
| *TtCarE-2* | Isoform0006669 | F:CAGGCTCTTCACCATCACCT<br>R:CGATCCCCATGTGAGTCGAG |
| *TtCarE-3* | Isoform0007569 | F:TTCTCGGAAGATTATTAGAAGGCAC<br>R:AACGCAGTCCATGCTCTGTT |
| *TtCarE-4* | Isoform0009688 | F:GCGTCTCCAAAGGAATCAGC<br>R:ACAAGAGCAGCTCCATGACC |
| *TtCarE-5* | Isoform0009763 | F:TTGGCCCTCTCGATGCAG<br>R:ACCAGAGCTGCAAGTTTCCT |
| *TtCarE-6* | Isoform0012624 | F:CTCATGTCAGGAGTTGCCGT<br>R:ACTAAGGGCAGCGAATGGTC |
| *TtCarE-7* | Isoform0013187 | F:TTTCGTCCATGGTTCTGGGT<br>R:TTACCAGGCATTGAAGTGGAAGA |
| *TtCarE-8* | Isoform0014318 | F:CAGAAGTGAGCGTCCTTGGT<br>R:ACTGGTTAACGGCCCAGAAG |
| *TtCarE-9* | Isoform0016197 | F:CGAGTTATTATTTTGCTGACGTACT<br>R:CGCTCCATAAAGTCCACCAGT |
| *TtCarE-10* | Isoform0016858 | F:AGGGAACCTCTTTTGGTTTCT<br>R:ACCGTGAACCTCATTGGTGT |
| *TtCarE-11* | Isoform0017046 | F:TGGAGAAGGCTGTTTCATCCC<br>R:ATGAATTGCTCGCAAAAGCCA |
| *TtCarE-12* | Isoform0017461 | F:ATCGCGCAACCAGCTTACAA<br>R:GCCCATTCACTGCAGCGTT |
| *TtCarE-13* | Isoform0017593 | F:GTCTAAAGTCGGCTGTGGCT<br>R:GAAAAGGTCGATCACTGGCG |
| *TtCarE-14* | Isoform0017690 | F:GTGAGATGCCAATGGTTGGTG<br>R:AAGCCCATTCGTTGTGGCA |
| *TtCarE-15* | Isoform0018315 | F:ACCTAGTCTCTGCTGTAATCGT<br>R:TGAGCGCTGATCGAAGTGAT |
| *TtCarE-16* | Isoform0018345 | F:ATGCCCACAAGATCCTTCCG<br>R:ACCAGAATTTCCTGCTCCCG |
| *TtCarE-17* | Isoform0018375 | F:GCTTTGACATTCGGCTTTGGA<br>R:GTTTGCTGGAATTCTCGGTTCTT |
| *TtCarE-18* | Isoform0019278 | F:AGCGTTCGAGCAGGTTTACA<br>R:TGGCATCCGAATTGCGTAGA |
| *TtCarE-19* | Isoform0020285 | F:TCAGCTACCTCCGATAGCACA<br>R:TGTGCCTTCTAATAATCTTCCGAGA |

Continue Table S1 Primers used for RT-qPCR and dsRNA synthesis.

| Gene name | Gene ID | Primer sequence (5'-3') |
|---|---|---|
| *TtCarE-20* | Isoform0020680 | F:TGGTCTCGTTTGCGGCACT<br>R:TCAACGGCTTTACCACACGAT |
| *TtCarE-21* | Isoform0020886 | F:AAGTTGACTTTGTTAATTTGCTTCG<br>R:CTGGTGCAGTTGCAAAAGGG |
| *TtCarE-22* | Isoform0023025 | F:ATGCCATTATGCAAAGTGGTTCA<br>R:AACCAGTTCTTCTGGGTGGTT |
| *TtCarE-23* | Isoform0025516 | F:AGCAACGGACATCAGGCTAA<br>R:GCCTCCCATCCTTCAGCAAG |
| *TtCarE-24* | Isoform0025977 | F:CTGTCAAAACCGGCAAAGCA<br>R:CCAGATGGATCGGTGAAGGG |

Table S2 Physicochemical characteristics of the 24 identified TtCarE proteins.

| Gene name | Molecular weight (kDa) | Isoelectric point (pI) | Number of amino acids (aa) | Signal peptide | Transmembrane domain | ORF length (bp) |
|---|---|---|---|---|---|---|
| *TtCarE-1* | 62.37 | 6.08 | 564 | None | 1 | 1692 |
| *TtCarE-2* | 118.59 | 6.41 | 1072 | None | 1 | 3216 |
| *TtCarE-3* | 88.50 | 8.53 | 784 | 1-21 aa | 1 | 2352 |
| *TtCarE-4* | 81.78 | 6.06 | 734 | None | 1 | 2202 |
| *TtCarE-5* | 83.46 | 5.79 | 761 | None | 1 | 2283 |
| *TtCarE-6* | 58.69 | 6.05 | 540 | None | 0 | 1620 |
| *TtCarE-7* | 52.63 | 5.24 | 472 | None | 0 | 1416 |
| *TtCarE-8* | 62.51 | 5.78 | 549 | None | 0 | 1647 |
| *TtCarE-9* | 68.79 | 8.62 | 624 | None | 0 | 1872 |
| *TtCarE-10* | 59.77 | 8.17 | 524 | None | 1 | 1572 |
| *TtCarE-11* | 60.52 | 8.55 | 539 | 1-20 aa | 0 | 1617 |
| *TtCarE-12* | 57.22 | 5.59 | 509 | None | 0 | 1527 |
| *TtCarE-13* | 62.27 | 5.87 | 562 | 1-22 aa | 0 | 1686 |
| *TtCarE-14* | 60.88 | 4.98 | 538 | 1-16 aa | 0 | 1614 |
| *TtCarE-15* | 62.68 | 6.21 | 551 | None | 0 | 1653 |
| *TtCarE-16* | 63.38 | 6.20 | 564 | 1-33 aa | 0 | 1692 |

Continue Table S2 Physicochemical characteristics of the 24 identified TtCarE proteins.

| Gene name | Molecular weight (kDa) | Isoelectric point (pI) | Number of amino acids (aa) | Signal peptide | Transmembrane domain | ORF length (bp) |
|---|---|---|---|---|---|---|
| *TtCarE-17* | 64.90 | 5.63 | 570 | None | 1 | 1710 |
| *TtCarE-18* | 35.58 | 5.40 | 313 | None | 0 | 939 |
| *TtCarE-19* | 50.40 | 6.32 | 458 | 1-21 aa | 0 | 1374 |
| *TtCarE-20* | 51.45 | 5.82 | 462 | 1-17 aa | 0 | 1386 |
| *TtCarE-21* | 51.42 | 5.90 | 462 | 1-17 aa | 0 | 1386 |
| *TtCarE-22* | 38.66 | 5.54 | 349 | None | 0 | 1047 |
| *TtCarE-23* | 34.21 | 5.70 | 309 | None | 0 | 927 |
| *TtCarE-24* | 30.05 | 6.00 | 261 | None | 0 | 783 |

Table S3 Predicted off-target analysis of *dsTtCarE-4* in target and non-target organisms.

| Gene name | Gene ID | Primer sequence (5’-3’) |
|---|---|---|
| *dsTtCarE-4* | Isoform0009688 | F:TAATACGACTCACTATAGGGTTGCGTCTCCAAAGGAATCAG<br>R:TAATACGACTCACTATAGGGGGGTTGGTATTTGGACGGA |
| *Actin* | | F:GCCATCCTTCGTTTGGATTTGGCT<br>R:TCTCGGACAATTTCTCGCTCAGCA |
| *RPS18* | | F:ACGTGCTGGTGAACTTACCGAAGA<br>R:TGCCTATTCAAGAACCAAAGTGGG |
| GFP | | F:TAATACGACTCACTATAGGGCAGTTCTTGTTGAATTAGATG<br>R:TAATACGACTCACTATAGGGTTTGGTTTGTCTCCCATGATG |

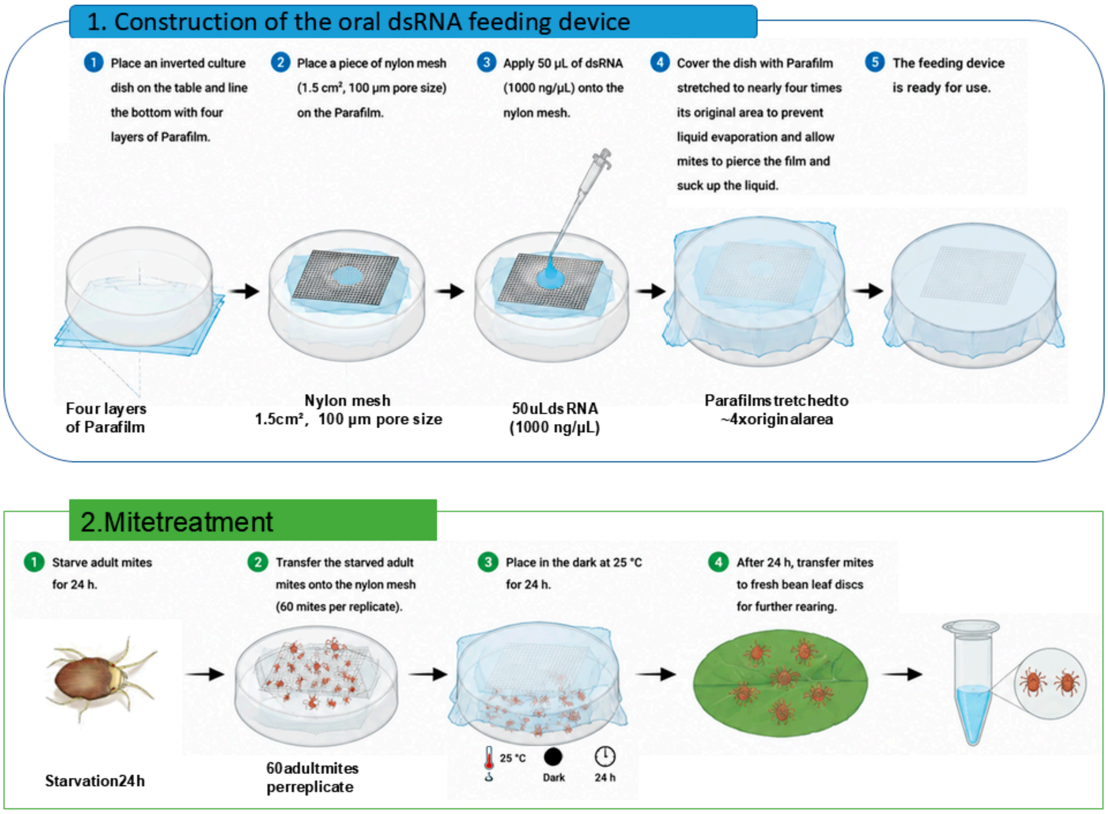


Figure S1. Experimental setup of the oral dsRNA feeding assay for *T. truncatus*.
